# The potentiality of bacteria to drive SARS-CoV-2 mutation

**DOI:** 10.1128/mbio.00539-24

**Published:** 2024-04-09

**Authors:** Xiangyu Zhang, Shun Li, Mengzhou Xue

**Affiliations:** 1 Department of Cerebrovascular Diseases, The Second Affiliated Hospital of Zhengzhou University, Zhengzhou, Henan, China; 2 Department of Immunology, School of Basic Medical Sciences, Chengdu Medical College, Chengdu, Sichuan, China; University of Calgary, Calgary, Canada

**Keywords:** SARS-CoV-2, bacteria, evolution

## Abstract

A recent study published in *mBio* by Cao et al. revealed the crucial roles of bacteria in benefitting SARS-CoV-2 mutations (B. Cao, X. Wang, W. Yin, Z. Gao, and B. Xia, mBio e3187-23, 2024, https://doi.org/10.1128/mbio.03187-23). Understanding the underlying mechanisms driving the evolution of SARS-CoV-2 is crucial for predicting the future trajectory of the COVID-19 pandemic and developing preventive and treatment strategies. This study provides important insights into the rapid and complex evolution of viruses facilitated by bacterial-virus interactions.

## COMMENTARY

The global spread and explosive development of the SARS-CoV-2 population have brought new mutational variability into the genome, allowing the virus to spread in front of growing population immunity while maintaining or boosting replication fitness (1, 2). The genetic diversity of SARS-CoV-2 (3, 4), along with the selective pressure exerted by antiviral drugs and vaccines (5, 6), has partially facilitated the emergence of dominant mutations in SARS-CoV-2. However, it is uncertain how SARS-CoV-2 accumulated numerous mutations rapidly, especially those providing an evolutionary benefit. Understanding the fundamental mechanisms that impact the evolution of advantageous mutations in SARS-CoV-2 will enhance our comprehension of these new mutations and the probable course of the COVID-19 pandemic. The human body harbors a large number of microbes, with approximately 90% of all human cells being linked to the microbiome (7). The human body sustains a thriving diversity of the microbiome, which forms a dynamic, auxiliary, functional system that evolves synergistically alongside its host’s physiological growth (8). The microbiome contributes an equal number of cells and significantly more genes to the human “holobiont,” and they have an impact on mammalian processes such as digestion, metabolism, immunity, and even brain function (9). However, before this study, even the most daring imagination would have yet to imagine that the human microbiome may facilitate the rapid acquisition of beneficial mutations by viruses.

In a study published in *mBio*, Cao et al. focus on understanding how the virus acquires a high number of beneficial mutations in a short time (10). The authors demonstrated that SARS-CoV-2 may collectively pick up mutations from the human microbiota that change the original viral binding sites or antigenic determinants, which results in variations of concern. The study by Cao et al. clarifies one of the possibilities of the evolving mutation mechanism of SARS-CoV-2. The authors developed a viral mutation fragment-based blast in the National Center for Biotechnology Information database to identify homologous fragments (HFs) harboring beneficial mutations with a frequency higher than 90%. Of the approximately 8,000 HFs obtained, 61 mutations in S proteins and other outer membrane proteins were found in the bacteria, accounting for 62% of all mutation sources, a 12-fold increase over the proportion of natural variation. Based on phylogeny and taxonomy theories, most of the bacterial species obtained in the blast were classified into four major bacterial phyla, namely, Proteobacteria, 41%; Actinobacteria, 29%; Bacteroidetes, 8%, and Firmicutes, 8%, consistent with the four major phyla of the human microbiota, although the proportions were different. Approximately 56% of the bacteria obtained were found in the lungs of healthy individuals, and 83% were also found in the human gut microbiota database, which led them to hypothesize that the human microbiota is a reservoir of homologous fragments. Therefore, the authors analyzed two gut microbe databases of COVID-19 patients (gutMEGA) and found that compared with the composition of non-COVID-19 gut microbes, the population of beneficial bacteria decreased significantly, while the number of harmful bacteria showed an increasing trend, which was similar to the change observed in the obtained bacteria carrying HFs (an increase in harmful Proteobacteria to 41% and a reduction in beneficial Firmicutes to 8%). In the intersection analysis with the bacteria containing S protein mutations, 15 of 55 mutations were detected in 19 differential bacterial genera in COVID-19 patients at the genus level, while 3 types of bacteria in COVID-19 patients were found to carry mutational HFs at the species level. These strong associations supported the hypothesis that SARS-CoV-2 might obtain HFs from the human microbiota.

To confirm whether viruses can use bacterial mRNA as an efficient template for extension to obtain mutations, the authors performed *in vitro* experiments using viral RNA-dependent RNA polymerase (RdRp) and synthetic short-stranded bacterial and viral mRNAs and found that the fusion of a bacterial mRNA (21–27nt) carrying mutations such as N501Y with a previously validated viral mRNA could be recognized by the RdRp and used as a template for extension, indicating that the exogenous bacterial mRNA was a valid template for the SARS-CoV-2 RdRp. Furthermore, the authors investigated whether complementary base pairs between bacterial mRNA and viral RNA could serve as “junction” sites to help the RdRp switch from viral RNA to bacterial mRNA, thereby using the bacterial mRNA as a template for extension. The results showed that despite mismatches between the viral primer and the bacterial mRNA template, two of the three mutations tested could be extended by RdRp to form chimeric viral-bacterial RNAs harboring mutations. These results suggest that viruses may introduce nucleotide mutations in bacterial mRNAs into their RNAs via RdRp during replication, resulting in beneficial mutations. These bacteria used as templates may originate from the human microbiota because of the extraordinarily high number of infections and the wide regions of infection, providing a large number of bacterial carriers for SARS-CoV-2. In summary, the authors presented the theory that HFs may have been acquired by SARS-CoV-2 from the human microbiome.

In conclusion, Cao et al. revealed a previously unexplored aspect of SARS-CoV-2 evolution: the acquisition of beneficial mutations from host bacteria. This study presents novel insights into the interaction of SARS-CoV-2 with host bacteria and elucidates the process of acquiring beneficial mutations from these host bacteria for the first time. This groundbreaking insight not only expands our understanding of viral evolutionary mechanisms but also complements existing theories of viral evolution, such as random mutation and stress screening. By elucidating the intricate interactions between viruses and host bacteria, including their role in mediating viral evolution, this study lays the foundation for the development of innovative prevention and treatment strategies targeting viral infections. For instance, insights gained from this study may inform the development of targeted antiviral therapies designed to disrupt viral evolution and transmission. In addition, this study offers possible explanations for puzzling phenomena, such as the effectiveness of antibiotics in treating viral infections, which may be related to the disruption of bacterial-viral interactions within the host. Additional studies are warranted to elucidate the mechanisms underlying the acquisition of bacterial genetic material by SARS-CoV-2 and its impact on viral phenotype and pathogenicity. Experimental studies using bacterial-viral co-infection models and longitudinal analyses of viral evolution in conjunction with the microbiome are essential to deepen our understanding of these complex interactions.
